# Regional water footprints of potential biofuel production in China

**DOI:** 10.1186/s13068-017-0778-0

**Published:** 2017-04-18

**Authors:** Xiaomin Xie, Tingting Zhang, Liming Wang, Zhen Huang

**Affiliations:** School of Mechanical Engineering, Shanghai Jiao Tong University, 800 Dongchuan RD. Minhang District, Shanghai, China

**Keywords:** Water footprints, Biofuels, China, Cassava, Sweet sorghum, *Jatropha curcas*

## Abstract

**Background:**

Development of biofuels is considered as one of the important ways to replace conventional fossil energy and mitigate climate change. However, rapid increase of biofuel production could cause other environmental concerns in China such as water stress. This study is intended to evaluate the life-cycle water footprints (WF) of biofuels derived from several potential non-edible feedstocks including cassava, sweet sorghum, and *Jatropha curcas* in China. Different water footprint types including blue water, green water, and grey water are considered in this study. Based on the estimated WF, water deprivation impact and water stress degree on local water environment are further analyzed for different regions in China.

**Results:**

On the basis of the feedstock resource availability, sweet sorghum, cassava, and *Jatropha curcas* seeds are considered as the likely feedstocks for biofuel production in China. The water footprint results show that the feedstock growth is the most water footprint intensive process, while the biofuel conversion and transportation contribute little to total water footprints. Water footprints vary significantly by region with climate and soil variations. The life-cycle water footprints of cassava ethanol, sweet sorghum ethanol, and *Jatropha curcas* seeds biodiesel were estimated to be 73.9–222.2, 115.9–210.4, and 64.7–182.3 L of water per MJ of biofuel, respectively. Grey water footprint dominates the life-cycle water footprint for each type of the biofuels. Development of biofuels without careful water resource management will exert significant impacts on local water resources. The water resource impacts vary significantly among regions. For example, based on blue and grey water consumption, Gansu province in China will suffer much higher water stress than other regions do due to limited available water resources and large amount of fertilizer use in that province. In term of blue water, Shandong province is shown with the most severe water stress issue, followed by Gansu province, which is attributed to the limited water resources in both provinces.

**Conclusions:**

By considering feedstock resource distribution, biofuel production potentials, and estimated water footprints, this study provides insight into the impact of biofuel production on the local water environment in China. Biofuel development policies need to be carefully designed for the sustainable development of biofuels in China.

## Background

Currently, China is the world’s largest energy-consuming country. The primary energy consumption in China accounted for 22.9% of the world’s total energy consumption in 2015 [1]. China faces major domestic and international challenges for secure energy supply and a balance between economic development and environment protection. Energy conservation and a low-carbon economy with significant greenhouse gas emission reductions are major strategic measures to deal with the challenges [2]. The Chinese government has set targets to reduce carbon dioxide (CO_2_) emissions per unit of gross domestic product (GDP) by 40–45% by 2020 and by 60–65% by 2030 [3], compared with the level in 2005. The development and utilization of renewable energy such as biofuels have been considered as the important ways to address energy security, greenhouse gas emissions, and other environmental issues in China [4].

Biomass can be transformed into gaseous, liquid and solid bioenergy, as well as other chemical materials and products [5]. Among these conversion technologies, liquid biofuels such as bioethanol and biodiesel are deemed as important substitutes for conventional petroleum fuels. Biofuels from different biomass feedstocks can be classified into four-generation biofuels [6]. First-generation biofuels are usually derived from edible feedstocks such as rice, wheat, sugar, and vegetable oils. Second-generation biofuels are produced mainly from non-food crops, non-edible vegetable oils, waste cooking oil, animal fat, crop residues, etc. Third-generation biofuels are referred to microalgae. And fourth-generation biofuels are from feedstocks such as industrial waste CO_2_ and other streams [6]. Each generation of biofuels has their advantages and disadvantages. For example, first generation biofuels can result in imbalance in the food supply and demand. Second generation biofuels are considered to be a suitable replacement to first generation biofuels since their feedstocks can be grown in marginal lands that are usually not suitable for crop cultivation. However, major issues of the second-generation biofuels include technology readiness, environmental sustainability, among other factors [7]. The commercial production of the third and fourth generation biofuels is yet to be demonstrated [8]. At present, the largest amount of biofuels produced worldwide are mainly from corn, sugarcane, soybean, rapeseeds, and other food crops [9].

Driven by various regulations, legislations, and plans that were adopted or proposed by Chinese government [10], the production of biofuels in China has increased considerably from four thousand tonnes of oil equivalent (Ttoe) in 2001 to 2430 Ttoe in 2015 [1], ranking China the fifth after the United States, Brazil, Germany, and France. Currently, the main feedstock for Chinese fuel ethanol is corn, used for 80% of the total domestic ethanol output [11]. However, due to the competition with food demand and the increase of grain prices [11], non-edible crops such as cassava, sweet sorghum, *Jatropha curcas* are considered to be preferred feedstocks for biofuels production.

Many studies have investigated the applicability of non-edible biofuels from the perspective of life-cycle energy consumption, economics, and environmental impacts such as greenhouse gas emissions, eutrophication, acidification, fresh water aquatic ecotoxicity, and human toxicity [12–29]. Over the past decade, some studies have examined the water footprint (WF) of biofuels [30–37], since the water consumption and agrochemical use in biofuel production could negatively impact both availability and quality of water resource [38].

Previous WF-related studies examined the WFs of different non-edible feedstocks, such as cassava, sweet sorghum, and *Jatropha curcas* (Table 1). The WF results of each biofuel pathway are shown with significant differences among studies due to different assumptions such as crop growth conditions, local climate, and crop management [39–42]. For example, Gerbens-Leenes et al. [43] provided a global overview of WFs of bioethanol from cassava and sorghum, and biodiesel from *Jatropha*. Within the study focusing on cassava-based ethanol, the water footprint of cassava ethanol was lower than these of sweet sorghum-based ethanol and *Jatropha*-based biodiesel, ranging from 783 to 2926 L water per L of ethanol. The WFs of sweet sorghum were shown with a range of 4394–13,541 L water per L of ethanol. The *Jatropha* biodiesel WFs from Gerbens-Leenes may be overestimated because of inappropriate use of data such as summing the rainfall and irrigation, but not of evapotranspiration [44]. Based on Jongschaap et al. [45], the WFs of *Jatropha* are 8281 L of water per L of *Jatropha* oil. The life-cycle WFs of *Jatropha* oil in Mozambique are reported to be as high as 15,264 L of water per L of *Jatropha* oil [46]. In China, the WFs of *Jatropha*-based biodiesel are estimated to be relatively low [40]. Generally, the water footprints of each biofuel show significant regional differences. Chiu’s study pointed out the importance to take regional-specific characteristics into consideration when implementing biofuel mandates [47].

**Table 1:** Summary of water footprints of biofuels in different regions

Biofuels	Feedstock	Region	Water footprint (L H_2_O/L biofuel)				Source
			Total	Blue	Green	Grey	
Bioethanol	Cassava	Global	2926	420	2506	–	[43]
		China	2827	22	520	2384	[40]
		Thailand	2414	703	1641	70	[39]
		Thailand	2161	6	2147	8	[41]
		Thailand	2021	335	1686	–	[48]
		Thailand	2582	449	2389	–	[49]
		Nigeria	783	407	376	–	[50]
	Sorghum	Global	9812	4254	5558	–	[43]
		Taiwan	4394	1740	2291	364	[42]
		China	13,541	35	1195	12,310	[40]
Biodiesel	*Jatropha curcas*	Mozambique^a^	15,264	3	15,261	–	[46]
		South Africa^a^	8281	–	–	–	[45]
		China	4565	23	845	3697	[40]
		Global^b^	19,924	11,636	8288	–	[43]

Conversion factors: Heating value—biodiesel: 37.7 MJ/kg [45], bioethanol: 29.7 MJ/kg; density—biodiesel: 0.88 kg/L; bioethanol: 0.7893 kg/L

^a^Only *Jatropha* oil is considered, not biodiesel

^b^Average values for five countries (India, Indonesia, Nicaragua, Brazil, and Guatemala)

Based on the International Energy Agency’s energy strategy scenarios for China, Cai et al. [51] evaluated the water withdrawal for energy production from 2011 to 2030. The results showed that the amount of water withdrawal would increase by 77% in 2030, which will aggravate China’s water scarcity risks under current energy strategy. Thus, addressing of water impacts of biofuel production in China is crucial for sustainable Chinese biofuel development.

The water resources of China are affected by both severe water shortage and serious water pollution. A large amount of toxic chemicals and industrial wastewater has been discharged into the rivers and groundwater [52]. Water pollution is widespread in China [53]. Agriculture sector is a major contributor to Chinese water pollution, due to the sector’s intensive fertilizer usage [54].

To examine water shortage issues, most past studies used the index of blue water footprint [36, 39, 43]. While green water footprint index is also used as to address life-cycle water footprint, many studies ignored grey water footprint [43, 45, 46, 50]. Zhang [40], Babel [39], Su [42], and Mangmeechai [41] evaluated the grey water of different biomass-based biofuels in China, Thailand, Taiwan, and Thailand, respectively. The grey water footprint is attributed to the fertilizer use. It is an indirect measure and could not reflect the actual water consumption. Hence, the impact of the grey water footprint requires further clarification for addressing the water quality issue. In this study, the impact of grey water footprint was evaluated.

The aim of this study is to evaluate the life-cycle water footprints of different non-edible biofuels in different regions in China on the basis of our previous studies [40]. In addition, the impact of future biofuel development on the local water stress is also assessed. To differentiate the impact of water use and water pollution, water deprivation potential (WDP) and water stress degree (WSD) are introduced in this paper to evaluate the potential water impacts of biofuels in different Chinese regions.

## Methods and data

### Development of biofuel production potential

The biofuel production potential for each non-edible biomass type is estimated using the following equation:

1 $$P_{\text{n}} = \mathop \sum \limits_{i}^{31} A_{i} \times Y_{i} \times C_{\text{n}}$$

where *P* refers to the production of each type of biofuels, in ton. *A* refers to the land area suitable for biomass cultivation, in hectare. *Y* means the average yield in each region for each biomass, in tons/hectare. *C* refers to the biofuel conversion rate. n means the type of biofuel, including bioethanol and biodiesel.* i* means each of the 31 regions in China.

Table 2 shows the yield of cassava, sweet sorghum, and *Jatropha curcas* in different regions in China [55–57]. For cassava, the data are from the field investigation in China, with the yield of 12–20 tons of fresh cassava per hectare and 7 tons of fresh cassava root (or 2.8 tons of dried cassava chips) to produce a ton of ethanol. The yield of sweet sorghum is affected by growing conditions. Okudoh et al. [58] pointed out that the yield of fresh sweet sorghum was only about 30 tons/hectare and with an ethanol conversion rate of 80 L per ton of sorghum in South Africa. The yield of fresh sweet sorghum stalk in China shows great regional differences ranging from 59.43 to 147.14 tons/hectare [59]. According to Zhao et al. [57], the average yield of dried sweet sorghum stem was about 14.5 tons/hectare in China, which accounted for 65% of the total aboveground dry matter. Based on site investigation, we assume that 16–18 tons of fresh sweet sorghum stem were required to produce a ton of ethanol. The yield of *Jatropha* seeds varies significantly from 0.3 to 12.5 tons of dry seeds/hectare due to climatic and soil conditions in different regions [60–64]. In this study, according to the production data in China, 5 [65] and 2.7 [64] tons/hectare *Jatropha* seeds are used for suitable land and less suitable land, respectively. For the biodiesel conversion rate, 2.9 tons of dried seeds are assumed for producing a ton of biodiesel [64].

**Table 2:** Yield for selected biomass types in China

Yield (tons/hectare)	Cassava root (fresh)	Sweet sorghum (fresh stem)	*Jatropha curcas* (dried seeds)
Guangxi	19.3	–	3.9
Guangdong	18.1	–	–
Yunnan	12.1	–	3.2
Fujian	16.4	–	–
Jiangxi	16.8	–	–
Heilongjiang	–	60.7 [66]	–
Jilin	–	79.0 [67]	–
Liaoning	–	68.4 [68]	–
Shandong	–	77.6 [67]	–
Gansu	–	79.3 [67]	–
Guizhou	–	–	2.7
Sichuan	–	–	3.0
Chongqing	–	–	2.7

### Water footprint estimation

**Fig. 1:**
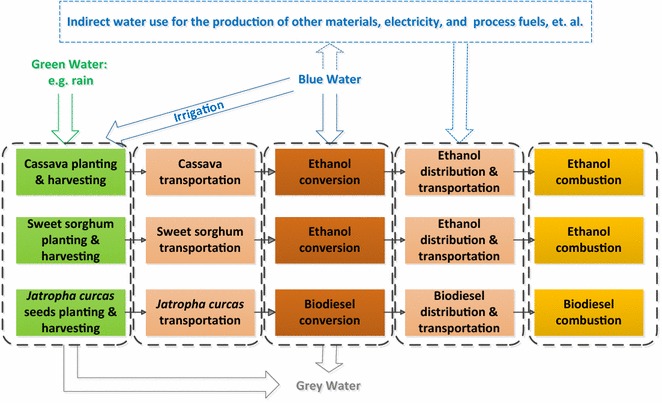
The system boundaries of the life-cycle water footprint

The methods used in this study to calculate the life-cycle water footprints for each biofuel pathway are based on Hoekstra’s method [30]. With Hoekstra’s method [30], green water footprint (WF_g_), blue water footprint (WF_b_), and grey water footprint (WF_gr_) are considered when calculating life-cycle water footprints. The blue water footprint includes the consumptive irrigation water lost through conveyance, operation, crop evapotranspiration (ET), and process water losses. Green water footprint refers to the rainfall amount lost through crop ET [29]. Grey water footprint is defined as the volume of freshwater that is required to assimilate the load of nutrients/chemicals to meet water quality standards [69]. Figure 1 shows the life-cycle analysis system boundaries for cassava, sweet sorghum, and *Jatropha curcas*-based biofuels, including feedstock growing, feedstock transport, biofuel production, biofuel transport, and biofuel utilization. Life-cycle water footprint is the sum of water footprint of all these stages. The functional unit is per MJ of biofuel produced and used.

#### Feedstock growth

The water footprint during biomass growing stage was estimated by Hoekstra’s method [30], in which blue water footprint (WF_b_, m^3^/ton) and green water footprint (WF_g_, m^3^/ton) are calculated from the crop water use (CWU, m^3^/hectare) divided by annual yield for each biomass. CROPWAT [70] model was used to estimate CWU for selected biomass from planting to harvesting in different regions. The CWU is calculated using the following equation:

2 $${\text{CWU}} = K_{\text{C}} \times {\text{ET}}_{0},$$

where $K_{\text{C}}$ is the crop coefficient which integrates the effect of characteristics that distinguish a specific crop from the Reference crop; ${\text{ET}}_{ 0}$ is reference crop evapotranspiration representing the potential evaporation of a well-watered grass crop. The Penman–Monteith equations [70] derived from Food and Agriculture Organization (FAO) were used to calculate the ${\text{ET}}_{ 0}$ value. The factors affecting ${\text{ET}}_{ 0}$ are climatic parameters including temperature, humidity, wind speed, sunshine hours, and solar radiation intensity. These climatic data plus rainfall in selected Chinese regions were obtained from China’s National Bureau of Statistics [71]. We choose the climatic data for year 2013 as a representative year in this study. The $K_{\text{C}}$ varies over the length of the growing period for different biomass feedstocks, which are summarized in Table 3. Soil data such as soil type and soil moisture were from the CROPWAT model [70]. Other data related to the CWU calculation are also listed in Table 3. The results of blue water and green water use for each crop in selected regions (information for regions selection in section “Results and discussion”) are derived from the model and presented in Table 4.

**Table 3:** Input parameters for the CROPWAT model for different biomass types in China

	Cassava [41]	Sweet sorghum	*Jatropha curcas* [46]
$K_{C}$ value, initial stage	0.3	0.3	0.6
$K_{C}$ value, mid-season	1.1	1.0	1.2
$K_{C}$ value, late season	0.5	0.55	0.4
Duration of initial stage (days)	60	20	20
Duration of development stage (days)	50	40	20
Duration of mid-season (days)	120	41	30
Duration of late season (days)	30	37	65
Rooting depth, initial stage (m)	0.2	0.3	0.3
Rooting depth, mid-season (m)	1.0	1.4	1.2

**Table 4:** Water use for each crop in selected Chinese regions

Index	Cassava					Sweet sorghum					*Jatropha curcas*				
	Guangxi	Guangdong	Yunnan	Fujian	Jiangxi	Heilongjiang	Jilin	Liaoning	Shandong	Gansu	Yunnan	Guizhou	Guangxi	Sichuan	Chongqing
ET_g_ (mm/d)	61.9	63.9	84.7	88.9	62.1	25	31.1	28	36.7	27.7	46.7	34.8	43.9	27.4	30.3
ET_b_ (mm/d)	16.5	12.2	61.3	27.6	4.5	10.8	13.9	4.6	22.7	18.6	26.8	10.7	11	13.5	3.84
CWU_g_ (m^3^/hectare)	967	998	1323	1389	970	391	486	437	573	432	730	544	686	428	473
CWU_b_ (m^3^/hectare)	258	191	958	431	70	169	217	72	355	291	419	167	172	211	60

*g* green water, *b* blue water

Grey water footprint of growing feedstocks was determined in accordance with Hoekstra et al. [30] as exhibited in Eq. (3). It is estimated in proportion of the fertilizer input in a region to the increased allowable pollution level. The allowable pollution level increase reflects the capacity of the ecosystem in the region to assimilate fertilizer loads.

3 $${\text{WF}}_{\text{gr}} = \frac{{\left( {\alpha \times {\text{AR}}} \right)/\left( {C_{\text{{max}} } - C_{\text{nat}} } \right)}}{Y}$$

In Eq. (3), WF_gr_ is grey water footprint in m^3^/ton; *α* is the leaching-run-off fraction; AR is the chemical application rate to the field per hectare, in kg/hectare; *C*_max_ is the maximum acceptable concentration in the ambient water stream, in kg/hm^3^; *C*_nat_ is the natural concentration for the pollutant considered, in kg/hectare; and Y is the crop yield, in ton/hectare. Since N fertilizer is the primary fertilizer used for feedstock growth, only N fertilizer is considered in estimating the grey water footprint. In this study, *α* and *C*_nat_ values are, respectively, 10% and 0 based on the literature [30]. In China, the surface water standard is classified into five grades. *C*_max_ refers to surface water Class V water standard [72]. Therefore, 0.2 mg/L is selected for N fertilizer as the maximum acceptable concentration in the ambient water stream. The amounts of N fertilizer use for growth of each biomass are summarized in Table 5.

**Table 5:** Nitrogen use for growth of each crop

N use (kg/hectare)	Cassava	Sweet sorghum	*Jatropha curcas*
Guangxi	187.5^a^	–	150.0 [73]
Guangdong	358.8 [74]	–	–
Yunnan	154.7^b^	–	25 [75]
Fujian	206.1^b^	–	–
Jiangxi	75.9^b^	–	–
Heilongjiang	–	136.8 [67]	–
Jilin	–	159.6 [67]	–
Liaoning	–	150.0 [67]	–
Shandong	–	120.0 [67]	–
Gansu	–	225.0 [67]	–
Guizhou	–	–	53.3 [76]
Sichuan	–	–	33.3 [77]
Chongqing	–	–	33.3^c^

^a^ From site investigation

^b^ Replaced with the average fertilizer use in China

^c^ Replaced with the data of Sichuan province

#### Biofuel conversion

The water consumption of biofuel plants was collected from these Chinese sources: one plant producing cassava-based ethanol, one plant producing sweet sorghum-based ethanol, and one producing *Jatropha curcas*-based biodiesel. Since the *Jatropha*-based biodiesel plant is still in demonstration phase, a regular biodiesel producing process was used to replace the *Jatropha*-based biodiesel conversion process. Because the water consumption among the three biofuel plants shows no obvious differences, the water consumption data are referenced from our previous study [40].

#### Feedstock and biofuel transportation

In this study, the water use for transportation of feedstocks from the fields to biofuel plants and transportation of biofuels from biofuel plants to refueling stations are combined together as the transportation stage. All of the transportation activities are assumed to be completed by truck with an average load of 18 tons and one was distance of 50 km. The amount of direct water use during biodiesel transportation and distribution is assumed to be 0.18 m^3^/ton of biofuel [78]. In this study, the WF of bioethanol transportation and distribution is assumed to be the same as biodiesel.

### Impact on local water resource

Comparing of water footprints of different biofuels alone does not reveal the actual water use burdens; the water stress at local levels needs to be taken into account [49]. To reveal the competitive pressure on water resources availability in a specific region, this study applies the water deprivation potential (WDP) approach [49] for the characterization factors to translate the impact of blue water for biofuel production in China. In addition, water stress degree (WSD) was also used to measure the water impact on local hydrologic system, since discharge of wastewater from different processes may cause different levels of potential pollution.

The steps to evaluate WDP index are described as following. First, the ratio of total water withdrawal to the gross amount of water resources is determined and expressed as “withdrawal-to-availability (WTA)” of the selected regions in China by using the data from the National Bureau of Statistics (NBSC) [55]. Second, the water stress index (WSI) with the equation in Pfister et al. [79] is determined. Finally, the potential water deprivation impact in a specific location is estimated as WDP = WF_b_ × WSI.

The water stress degree (WSD) is defined as the sum of blue and grey WF or blue water WF in each region divided by local total water resource. Two sets of results were produced in this study. One considered both blue and grey water footprint, and the other considered only blue water footprint. For illustrative purposes, WSDs are categorized into five levels including extreme, severe, stress, moderate, and low according to Gheewala et al. method [49]. In this study, 0–1% refers to level low, 1–2% refers to level moderate, 2–4% refers to level stress, 4–6% refers to level severe, and >6% refers to level extreme.

### Biomass resource distribution

China has plenty of biomass resources such as agricultural residues, forest residues, and animal manures. The priorities of energy development in China are utilization of marginal land resources, selection and cultivation of energy biomass feedstock species, and efficient utilization of waste energy [80]. The potential of agricultural residues, forest residues, and animal manures in China are 748.16, 104.5, and 922.3 Mt, respectively [81]. It is projected that about 117.85 Mt of bioethanol and 34.28 Mt of biodiesel could be produced from these potential resources in 2030 [82].

**Fig. 2:**
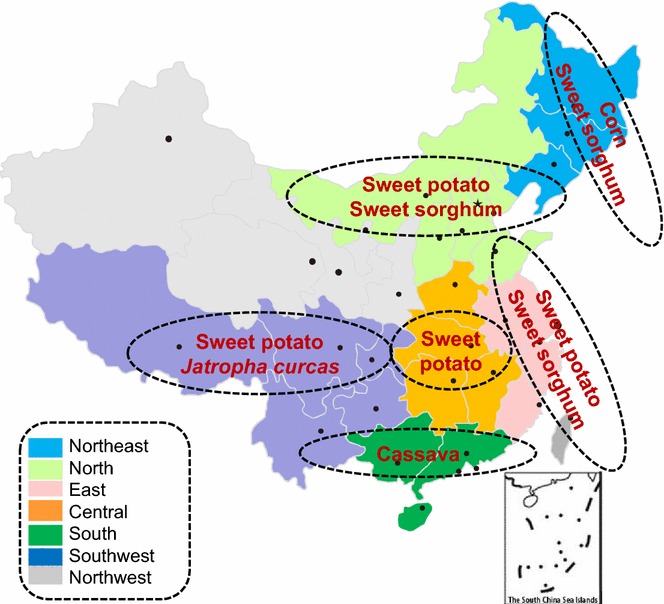
The six future non-edible biofuel production regions in China

In China, popular biofuel feedstocks include sugar beet, sugarcane, tuber crops, sweet sorghum, *Jatropha curcas*, among others. The distribution of the non-edible biofuels is shown in Fig. 2. Among these crops, cassava, sweet sorghum, and *Jatropha curcas* are considered as the likely feedstocks for producing biofuels. According to the data from China Rural Statistical Yearbook and China Statistical Yearbook, the total amount of these biomass feedstocks were 14.68 Mt in 2015 [55, 56]. These bioenergy crops are primarily concentrated in southwest, south, and northeast China. North and northeast regions have large sweet sorghum production potentials. Vast areas in Guangxi, Yunnan, and Guangdong provinces are available for planting cassavas. Guangxi, Guizhou, and Henan have plenty of *Jatropha curcas* potential.

In addition to the 135 million hectare of arable land used to ensure the nation’s grain production [52], the available non-arable land areas in China are still very large. According to a survey [80], China has 108 million hectares of uncultivated lands, and 35.35 million hectares of which are suitable for agriculture, accounting for 32.7% of the total marginal area. The total marginal land may be equivalent to 36.9% of the existing arable land area. Forestry land covers 253 million hectares [52], but only 76.62 million hectares of marginal mountains and lands are suitable for tree planting, accounting for 28.6% of the woodland area. Considering crop ecological adaptability, the marginal areas suitable for planting sweet sorghum, cassava, and sugar cane in China are approximately 13, 5, and 15 million hectares, respectively [80].

## Results and discussion

### Biofuel production potential

**Fig. 3:**
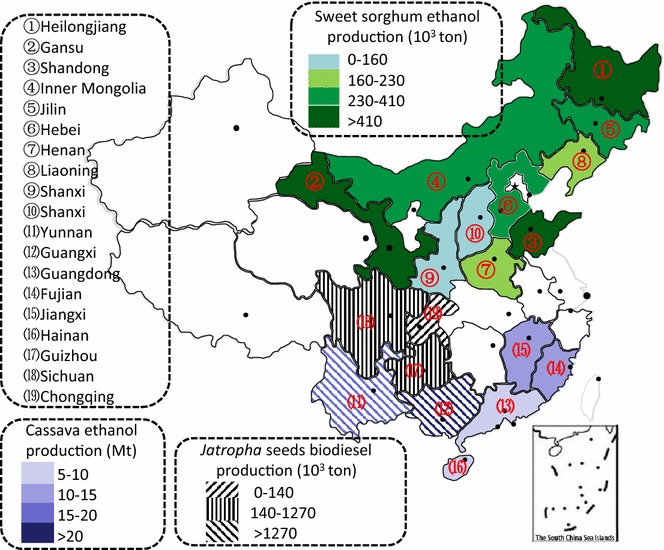
The production potentials of non-edible biofuels in China

The development of biofuels is highly dependent on the technical efficiency of the agriculture system and the associated social and ecological benefits of biofuels in a country. The available land resources and the production efficiency per unit of land area are the two main factors affecting the biofuel production potential. Based on Eq. (1), biofuel production potentials from non-edible biomass in different regions in China were assessed and are show in Fig. 3.

#### Sweet sorghum ethanol

In Fig. 3, for bioethanol production potentials from sweet sorghums, the northeast region is the most suitable place for sweet sorghum ethanol production. 59.19 million hectares of unused lands are available for sweet sorghum production, mainly located in Xinjiang and Inner Mongolia regions. The most suitable areas for the production cover about 2.87 million hectares. Those areas are mainly distributed in Inner Mongolia, Heilongjiang, Shandong, and Jilin provinces with favorable conditions such as rainfall, soil fertility, and land slopes [83]. The ethanol production potential from sweet sorghum could reach more than 118.39 Mt from unused lands in general, and 5.73–26.38 Mt from the most suitable unused lands, with an average of 10.75 Mt. This amount of ethanol production could meet 84.8% of the demand for blending 20% ethanol in Chinese gasoline. To promote commercial-scale production of sweet-sorghum-based ethanol, some demonstration projects have been carried out in Heilongjiang, Xinjiang, Shandong, Inner Mongolia, and Liaoning provinces in China.

#### Cassava ethanol

As shown in Fig. 3, the cassava production potential is in southwestern China. The suitable regions for cassava planting are mainly distributed in southern provinces such as Guangxi, Yunnan, Fujian, Guangdong, Jiangxi, and Hainan provinces. Guangxi has the highest potential. Guizhou, Hunan, Chongqing, and Zhejiang provinces show some potential. However, the suitable but unused lands for cassava planting in these regions cover some limited areas, only 1.53 × 10^4^ hectares, and the cassava ethanol production potential from these lands could only satisfy 1.0% of the China’s total demand for E10 gasoline market [84].

#### *Jatropha curcas* biodiesel

Biodiesel, derived from vegetable oil, animal fats, algal lipids, or waste grease through “trans esterification” in the presence of alcohol and alkaline catalyst, has been commercially produced and used as a petroleum diesel substitute [85]. Chinese government sets a goal to produce 12 Mt biodiesel annually by 2030 [86]. At present, biodiesel production in China is still in infancy with a total annual capacity of 5 Mt [11].

As a biodiesel feedstock, production of oil seeds from *Jatropha curcas* is widely distributed from dry subtropical regions to tropical rain forests in China. Its production areas are mainly in Guangdong, Guangxi, Yunnan, Sichuan, Guizhou, Taiwan, Fujian, and Hainan Provinces. Other tropical and subtropical regions are also potentially suitable for the plant [87]. Based on the potential land and conditions such as temperature, moisture, gradient, and soil for *Jatropha curcas* growth, the suitable land areas are found in three main producing regions, as listed in Table 6. In particular, large areas of available lands in Guangxi and Yunnan provinces are suitable for *Jatropha curcas* growing.

**Table 6:** Land suitable for *Jatropha curcas* planting in China (10^3^ hectare) [88, 89]

Land classification	Guangxi		Yunnan		Guizhou	
	Suitable	Less suitable	Suitable	Less suitable	Suitable	Less suitable
Open forest land	1153	1378	53.0	814.2	0.2	290.3
High coverage grassland	503.1	703	126.2	1085.1	0	1.7
Moderate coverage grassland	81.6	97.9	32.2	443.9	9.3	244
Low coverage grassland	2.5	5.9	5.7	27.7	0.5	29.1
Beaches	1.5	0	0	0	0	0
Beachland	10	5.4	1.2	4.5	0	0
Bare land	0.9	0		1.5	0	0
Suitable land for *Jatropha*	1249.5	1487.2	92.1	1291.8	10.0	563.4
Modified area for *Jatropha*	999.6	1189.8	73.7	1033.4	8.0	450.7

Figure 3 also shows the biodiesel production potential from *Jatropha curcas*. The *Jatropha curcas* seed yield can reach 9.75 ton/hectare with 40% oil content based on dry mass [87]. In this study, we assumed that the average yield of *Jatropha curcas* seeds in suitable and less suitable land are approximately 5 [65] and 2.7 [64] tons/hectare, respectively. Based on the figures, Yunnan and Guangxi provinces show the highest biodiesel potential from *Jatropha curcas* seeds, followed by Guizhou, Sichuan, and Chongqing provinces.

### Life-cycle water footprints of biofuels

#### Results for different production processes

**Fig. 4:**
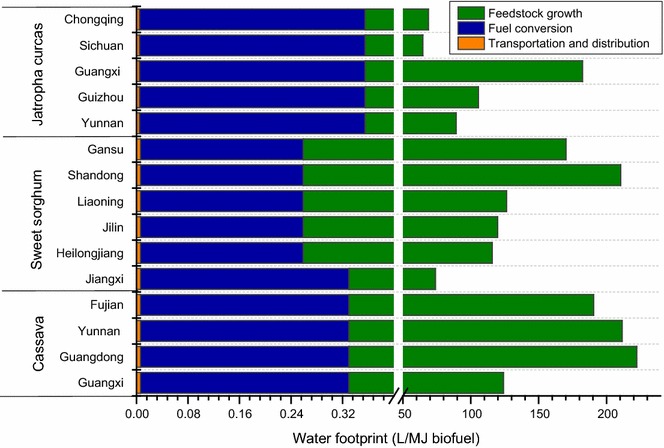
Life-cycle water footprints of biomass-based biofuels

Figure 4 shows the life-cycle water footprints of the three biofuel pathways in different regions in China: cassava-based ethanol, sweet sorghum-based ethanol, and *Jatropha curcas* seed-based biodiesel. Here, life-cycle water footprints include blue, green, and grey water footprint. Feedstock planting stage contributes 99.5–99.9% of the total life-cycle water footprint for each biofuel, while the WFs of transportation stage and biofuel conversion stage are 0.004–0.006 L/MJ biofuel and 0.25–0.35 L/MJ biofuel, respectively. This is because growing of the biomass requires large amount of water covering direct water and indirect water consumption. Direct water consumption includes green water footprint such as rainfall and blue water such as irrigation water. Indirect water consumption refers to the grey water caused by the use of fertilizer. Similarly, many other studies showed that crop growing stage dominated the total life-cycle water footprint [35, 90, 91].

The water footprints of the three biofuel pathways are significantly different. Life-cycle WFs for cassava-based ethanol, sweet sorghum-based ethanol, and *Jatropha curcas*-based biodiesel are 73.9–222.2 L/MJ ethanol, 115.9–210.4 L/MJ ethanol, and 64.7–182.3 L/MJ biodiesel, respectively. Cassava-based ethanol in Guangdong province shows the largest water footprint, followed by cassava ethanol in Yunnan province, sweet sorghum-based ethanol in Shandong province, and *Jatropha curcas* seed-based biodiesel in Guangxi province. Compared to sweet sorghum and cassava, *Jatropha curcas* shows relatively low WF. It is demonstrated that the average water consumption rate of *Jatropha* is 6 L per week [62] throughout the growing season, which means that *Jatropha* can survive and produce full yield with high-quality seeds under minimum water requirements.

Guangdong province ranked the first in the water footprints of cassava ethanol, followed by Yunnan, Fujian, Guangxi, and Jiangxi provinces. For sweet sorghum-based ethanol, Shandong province shows the largest water footprints, followed by Gansu, Liaoning, Jilin, and Heilongjiang provinces. For *Jatropha curcas*-based biodiesel, Guangxi province ranks the first in water footprints, followed by Guizhou, Yunnan, Chongqing, and Sichuan provinces. The regional differences of the water footprint for specific biofuel pathways are attributed to different local conditions such as climate, crop yield, and crops management. For example, the soil in Gansu province is relatively poor [67], compared with other regions like Jilin province, so more fertilizer is required to improve the yield of sweet sorghum. In addition, extra irrigation water also contributes to higher WFs in the arid regions.

#### Results for different water footprint types

**Fig. 5:**
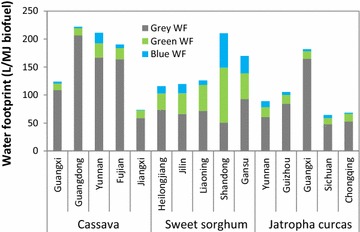
Life-cycle water footprint by different water types

Figure 5 shows the water footprint by different water types for each fuel pathway. It is obvious that the grey water accounts for the largest proportion of the total water footprints of biofuels. This is due to the fertilizer use for feedstock growth. The larger the amount of applied fertilizer, the higher grey water footprints. The grey water for sweet sorghum shows no significant difference among regions. Compared to sweet sorghum, the grey water for cassava and *Jatropha curcas* is relatively high. This is attributed to the amount of fertilizer applied. Green water footprint is also an important contribution to the total water footprint for each biofuel. Sweet sorghum ethanol shows larger green water footprint than cassava ethanol and *Jatropha curcas* biodiesel. The green water is connected with crop features and climate conditions, such as crop height, soil conditions, and rainfall. As for the blue water, sweet sorghum also shows larger blue water footprint than cassava and *Jatropha curcas.* This is because sweet sorghum needs a large amount of irrigation water, especially in the arid areas like Gansu and Shandong provinces. Additionally, cassava also needs certain irrigation in dry areas such as Yunnan province. In these arid regions, irrigation is required to compensate for the water need by evapotranspiration. In addition, the yield of the feedstock also causes the differences of water footprint for each biofuels pathway.

### Water stress on local water environment

#### Water deprivation impact potentials in China

The WSI values of the selected Chinese regions are estimated as the characterization factors to show the water deprivation potential. To calculate this indicator, blue water consumption for biofuel production in a specific region was multiplied with the WSI of that region and presented in L water per MJ biofuel. Table 7 presents the related WTA, WSI, and WDP values. The results show that the WSI values in China vary significantly by region, ranging from 0.02 in southwestern region to 0.99 in Shandong province. The higher the WSI values, the greater impacts on local water resources. Table 7 also shows the water deprivation potentials (WDP) from the consumptive water to produce one MJ of biofuel in different regions. The results reveal that the WDP indicator can help screen and prioritize the areas that potentially face significant water competition, which cannot be revealed by the WF values. For instance, cassava ethanol produced in Guangdong province; sweet sorghum-based ethanol produced in Shandong, Liaoning, and Gansu provinces; and *Jatropha curcas* seed-based biodiesel produced in Yunnan province could result in greater impacts of the water deprivation than in other selected regions.

**Table 7:** Water deprivation potentials in China

	WTA	WSI	WDP (L/MJ biofuel)		
			Cassava	Sweet sorghum	*Jatropha curcas*
Guangxi	0.15	0.03	0.09	–	0.09
Guangdong	0.26	0.05	0.12	–	–
Yunnan	0.09	0.02	0.32	–	0.18
Fujian	0.17	0.03	0.18	–	–
Jiangxi	0.16	0.03	0.03	–	–
Heilongjiang	0.39	0.11	–	1.36	–
Jilin	0.43	0.14	–	2.32	–
Liaoning	0.97	0.84	–	6.41	–
Shandong	1.45	0.99	–	60.44	–
Gansu	0.61	0.33	–	10.25	–
Guizhou	0.08	0.02	–	–	0.08
Sichuan	0.09	0.02	–	–	0.10
Chongqing	0.13	0.02	–	–	0.04

WDP here only relates to blue water

#### Water stress degree on local water environment in 2030

To further evaluate the impact of the future biofuel production on local water resources, we predicted the water stress degree (WSD) in 2030 based on the prediction of biofuel production in 13 selected regions in China. The biofuel production in 2030 in the selected regions was estimated based on the biofuel development goal in 2030, the average growth rate of each biofuel, and available land for growing feedstock in each region. Table 8 lists the biofuel production prediction in 2030 in different regions. The production in 2030 was extrapolated through average growth rate on the basis of the output of recent years [55, 56].

**Table 8:** Biofuel production prediction in 2030

Production (10^3^ ton)	Cassava ethanol	Sweet sorghum ethanol	*Jatropha curcas* biodiesel
Guangxi	468	–	844
Guangdong	393	–	–
Yunnan	349	–	250
Fujian	361	–	–
Jiangxi	161	–	–
Heilongjiang	–	259	–
Jilin	–	543	–
Liaoning	–	350	–
Shandong	–	291	–
Gansu	–	651	–
Guizhou	–	–	94
Sichuan	–	–	100
Chongqing	–	–	50

**Fig. 6:**
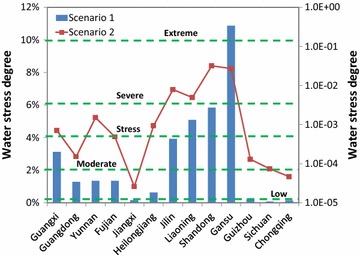
The water stress degree due to non-edible biofuel development in China (Scenario 1 is shown in the *left y-axis*; Scenario 2 is shown in the *right y-axis*)

Figure 6 shows the predicted results of WSDs in 2030. Results for two scenarios are presented. In Scenario 1, the sum of blue and grey WF were used to estimate the WSD, while in Scenario 2, only blue WF was considered to estimate the potential effect on local water resources.

The WSD in Scenario 1 showed much higher than that of Scenario 2, which indicates that the excessive use of fertilizer does have significant impact on local water resources. For example, in Scenario 1, the WSDs in the four southwest provinces including Sichuan, Chongqing, Jiangxi, and Guizhou provinces are very low, with a range of 0.07–0.24%. In Guangdong, Yunnan, Fujian, and Heilongjiang provinces, the WSDs are in the moderate level. Jilin and Guangxi provinces obviously have water stress problems. Liaoning and Shandong provinces face severe water stress problems too. The WSD in Gansu province is more than 10%, which shows extreme water stress on local water resources. In Scenario 2, Shandong province shows the highest WSD of 3.2%, followed by Gansu province with a value of 2.7%. Jilin and Liaoning provinces show some stress, while the rest of selected provinces face relative low WSD.

Water stress degree is driven by these factors: (1) future production volume of biofuels; (2) growing conditions of crops in each region; (3) crop management for each biomass feedstock in different regions; and (4) local available resources. For example, on the one hand, the total water resources in Gansu, Shandong, and Liaoning provinces are <20 billion m^3^. On the other hand, these regions have abundant available unused lands for producing biofuels in the future. Therefore, these regions will face extreme water stress if the development of biofuels is based on land availability.

In order to reduce water footprints and mitigate water shortage, the development of biofuel requires well-organized management. Take Thailand for example, with proper management, such as reducing irrigation, reducing chemical fertilizer use, and using cassava chips, the water footprint of biofuels in Thailand could be reduced by at least 53%, or 1.33 × 10^10^ m^3^, annually [92].

## Conclusions and policy recommendations

In this study, the biofuel production potential from different non-edible biomasses was estimated in China. With this, regional water footprints of cassava-based ethanol, sweet sorghum-based ethanol, and *Jatropha curcas* seed-based biodiesel were evaluated from the life-cycle perspective. Moreover, the water stresses with large-scale development of biofuels in the future were also examined.

The regional production potential results showed that southwest China is suitable for cassava-based ethanol production and *Jatropha curcas* seed-based biodiesel production, while northeast China shows significant potential for sweet sorghum-based ethanol production. The life-cycle water footprint of cassava-based ethanol, sweet sorghum-based ethanol, and *Jatropha curcas* seed-based biodiesel are 73.9–222.2, 115.9–210.4, and 64.7–182.3 L/MJ, respectively. Compared with cassava-based ethanol and *Jatropha curcas*-based biodiesel, sweet sorghum-based ethanol showed the relatively lower water footprint. Grey water dominated the life-cycle water footprint. The water footprint results for each biofuel pathway vary significantly by region. The regional differences of the water footprint for a specific biofuel pathway are attributed to local conditions such as climate, crop yield, and crop management.

Production of biofuels will certainly have impacts on local water resources. Cassava-based ethanol production in Yunnan province, sweet sorghum-based ethanol production in Shandong province, *Jatropha curcas* seed-based biodiesel produced in Yunnan province will result in the water deprivation impact greater than in other regions. From the view of blue water consumption, the water stress degree results in Shandong province showed extreme water stress on local water resources, followed by Gansu province. While from both the blue and grey water footprint, Gansu province had the extreme water stress degree, followed by Shandong, Liaoning, and Jilin provinces.

With the increased demand for energy in China, the availability and quality of water may constrain the Chinese capability to improve its energy security through alternative fuels with high water footprints. Rational development policies and well-designed management are needed to ensure sustainable development of non-edible biofuels. From the results in this study, we made the following recommendations for Chinese biofuel development. First, the appropriate development scale of each biofuel type needs to be established according to local conditions including water supply and demand. Second, fertilizers have played an important role in increasing crop productivity. However, excessive use of fertilizers has already caused adverse environmental effects. To reduce these effects and especially water quality effects, fertilizer use should be controlled as much as possible. Finally, the large amount of wastewater discharge is a major barrier for the development of biofuels. Thus, adequate treatment of wastewater from biofuel facilities is key to sustainable biofuel development in China.

## Abbreviations

Ttoe: thousand tonnes of oil equivalent
WF: water footprint
bn: billion
gha: global hectares
WDP: water deprivation potential
WSD: water stress degree
WTA: withdrawal-to-availability
WSI: water stress index
WF_b_: blue water footprint
WF_g_: green water footprint
Wf_gy_: grey water footprint
